# IMOS National Reference Stations: A Continental-Wide Physical, Chemical and Biological Coastal Observing System

**DOI:** 10.1371/journal.pone.0113652

**Published:** 2014-12-17

**Authors:** Tim P. Lynch, Elisabetta B. Morello, Karen Evans, Anthony J. Richardson, Wayne Rochester, Craig R. Steinberg, Moninya Roughan, Peter Thompson, John F. Middleton, Ming Feng, Robert Sherrington, Vittorio Brando, Bronte Tilbrook, Ken Ridgway, Simon Allen, Peter Doherty, Katherine Hill, Tim C. Moltmann

**Affiliations:** 1 CSIRO Oceans and Atmosphere, GPO Box 1538, Hobart, Tasmania 7001, Australia; 2 CSIRO Oceans and Atmosphere, 41 Boggo Rd, Dutton Park, Queensland, 4102, Australia; 3 Centre for Applications in Natural Resource Mathematics, University of Queensland, St. Lucia, Queensland 4072, Australia; 4 Australian Institute of Marine Science, PMB #3, Townsville MC, Queensland 4810, Australia; 5 School of Mathematics, University of New South Wales, Sydney, New South Wales 2052, Australia; 6 South Australian Research and Development Institute, PO Box, 120 Henley Beach, South Australia 5022, Australia; 7 CSIRO Oceans and Atmosphere, Floreat, Western Australia 6014, Australia; 8 CSIRO Land and Water, Clunies Ross St, Black Mountain, Australian Capital Territory 2601, Australia; 9 Integrated Marine Observing System, University of Tasmania, Private Bag 110, Hobart, Tasmania 7001, Australia; University of Vigo, Spain

## Abstract

Sustained observations allow for the tracking of change in oceanography and ecosystems, however, these are rare, particularly for the Southern Hemisphere. To address this in part, the Australian Integrated Marine Observing System (IMOS) implemented a network of nine National Reference Stations (NRS). The network builds on one long-term location, where monthly water sampling has been sustained since the 1940s and two others that commenced in the 1950s. *In-situ* continuously moored sensors and an enhanced monthly water sampling regime now collect more than 50 data streams. Building on sampling for temperature, salinity and nutrients, the network now observes dissolved oxygen, carbon, turbidity, currents, chlorophyll *a* and both phytoplankton and zooplankton. Additional parameters for studies of ocean acidification and bio-optics are collected at a sub-set of sites and all data is made freely and publically available. Our preliminary results demonstrate increased utility to observe extreme events, such as marine heat waves and coastal flooding; rare events, such as plankton blooms; and have, for the first time, allowed for consistent continental scale sampling and analysis of coastal zooplankton and phytoplankton communities. Independent water sampling allows for cross validation of the deployed sensors for quality control of data that now continuously tracks daily, seasonal and annual variation. The NRS will provide multi-decadal time series, against which more spatially replicated short-term studies can be referenced, models and remote sensing products validated, and improvements made to our understanding of how large-scale, long-term change and variability in the global ocean are affecting Australia's coastal seas and ecosystems. The NRS network provides an example of how a continental scaled observing systems can be developed to collect observations that integrate across physics, chemistry and biology.

## Introduction

Human activities are intricately linked to coastal marine systems both directly and indirectly [1]. Multiple drivers such as climate, pollution and exploitation that vary in intensity and both spatial and temporal distributions are played out in the coastal sphere [2] and changes to coastal systems have flow-on effects. These including increased susceptibility of coastal communities to sea level change and floods, loss of ecosystem services and erosion [3].

To detect and predict these impacts, the Global Ocean Observing System (GOOS) advocates sustained, routine and reliable observations on local, regional and global scales [4], to define short-term variability through longer-term change in system and ecosystem response [5]. These observations should be collected in a way that allow the provision of information on time scales required for informed decision making by local, regional and national management agencies [6].

The increased understanding of the role of the oceans in determining the global climate system has been accompanied by a growing realisation that, in comparison to terrestrial systems, sustained (> 10 yr) marine observations, particularly in the Southern Hemisphere, are sparse [5], [7]. Offshore physical oceanography has been well served by new technology and internationally integrated platforms such as the Array for Real-time Geostrophic Oceanography (ARGO) [8], [9]. Broad scale observing of coastal seas and sustained biological observations, however, are still relatively data poor [10].

There are considerable challenges in achieving observations in coastal seas. Satellite remote sensors for ocean properties can degrade in their spatial and optical resolution close to land masses, requiring *in situ* validation and characterization. *In situ* systems, however, need to be robust to meet challenges associated with the dynamic nature of the coastal environment; such as high wave energy, turbidity, fouling and environmental extremes. There are also many challenges associated with the integration of data required for regional and national syntheses. These include access and management of data, development of pathways to promote collaboration and ongoing support for the continued collection of those data.

In Australia, logistics present additional challenges in the establishment of sustained observing. Australia has the third largest ocean territory in the world and a continental coastline of 36,000 km, which increases to 60,000 km when the nation's numerous offshore islands are also considered [11]. As well, though the geographic size of Australia is similar to that of the continental United States of America, Australia's population, at approximately 23 million people [12], is an order of magnitude smaller.

**Figure 1:**
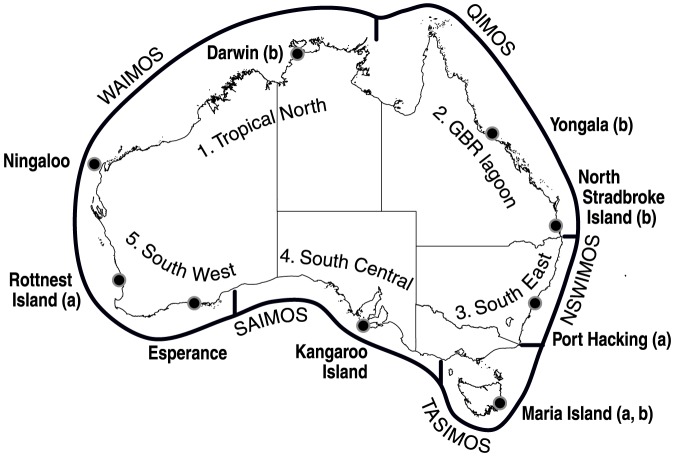
Locations of the nine National Reference Stations in relation to the five coastal IMOS node regions and the five general environmental planning regions; a: long term site; b. site with telemetry.

Considering these constraints, a national and co-ordinated approach was adopted by Australian federal and state governments to address a general lack of long-term and geographically comprehensive oceanographic monitoring. In 2007, the Integrated Marine Observing System (IMOS) was established under an unprecedented partnership between Australia's major marine research institutes [13]. IMOS has been designed in line with GOOS principles [14] and includes linked global (ocean basin/climate) and coastal components. Guided by these principles, researchers contributing to IMOS are organized into six science nodes consisting of a blue water and climate observing node and five regional coastal nodes (Fig. 1). The nodes (which have in excess of 600 members in total) have developed science plans (Available at www.imos.org.au/nodes) aimed at addressing five major research themes: 1) multi-decadal ocean change; 2) climate variability and weather extremes; 3) major boundary currents and inter-basin flows; 4) continental shelf processes and 5) biological responses. The science plans are implemented by ten facilities that deploy equipment and deliver data to an eleventh facility, the Electronic Marine Information Institute (eMII), which archives and facilitating public access to data and metadata. The Australian National Mooring Network (ANMN) is the largest of the IMOS facilities and maintains a network of National Reference Stations (NRS) and associated regional moored sensor arrays that monitor Australia's coast and continental shelf oceanography [15].

Prior to the advent of IMOS, long-term observation in Australia reflected the global trend of rare and failed programs [16], [17]. Of 42 national coastal stations operated between 1942 to the present day (Source: CSIRO Marlin database) only four with greater than 20 years data are still operational: two at Port Hacking in New South Wales at 50 m and 100 m (established in 1942 and 1953, respectively), one at Maria Island on Tasmania's east coast (established in 1944) and one at Rottnest Island in Western Australia (established in 1951) (Fig. 1). Data collected at these stations commenced with salinity and temperature and evolved to include nutrients (silicate, nitrates/nitrites, phosphate) and dissolved oxygen at Maria [18] and are considered globally significant, as there are few other multi-decadal time series in coastal areas of the Southern Hemisphere. Datasets generated by these long term stations have been used to identify climate change signals in Australia's two principle boundary currents, the East Australian Current (EAC) [19] and the Leeuwin Current [20]. The time-series data provided by the stations have also been used as references to understanding changes in biological systems associated with these currents, such as latitudinal shifts in temperate fish and kelp distributions [21], [22] and long term changes in phytoplankton growth rates and biomass [18].

The expanded NRS network commenced its roll-out in 2008 and was designed to build upon the existing time-series stations, to ensure their maintenance and to expand to cover other marine provinces around Australia's coastal seas. The aim of this paper is to document the rationale, methods, logistics, data collection, and configuration of the NRS and provide key examples of data to demonstrate enhanced utility and highlight the science opportunities that this free and open access system affords. The NRS provides a *modus operandi* or a blueprint for future continental or other large-scale coastal observing systems globally, helping to standardize observing efforts and optimize the use of resources.

## Methods

### The scientific rationale

The rationale of the NRS is to: 1) provide a multi-disciplinary and integrated suite of time-series observations for reference by more spatially-distributed and intensive shorter-term studies; 2) to establish a coastal information infrastructure through the development of national data standards; and 3) make available *in-situ* measures for the validation of modeling activities and coastal remote sensing. Regionally, individual NRS act as focal points for the integration of datasets collected by other IMOS operations such as the continental shelf mooring arrays of the ANMN, ships of opportunity, ocean gliders, autonomous underwater vehicles, ocean radar and animal-borne tags, to allow for the investigation of linkages between coastal and offshore environmental systems and processes.

### Location and number of stations in the network

For the purposes of NRS planning and implementation, ‘coastal ocean’ was defined as waters from the high tide mark to the shelf break (∼200 m isobath). Taking into account the location of the existing historical stations, selection of the number and location of additional stations required to form the expanded NRS network was based on four criteria: 1) the location of principal currents in the Australian coastal region; 2) the distribution of Australian coastal phytoplankton provinces; 3) information requirements for monitoring a national network of marine reserves; and 4) regions of priority for each of the six IMOS science nodes.

#### Principal currents of the Australian coastal region

**Figure 2:**
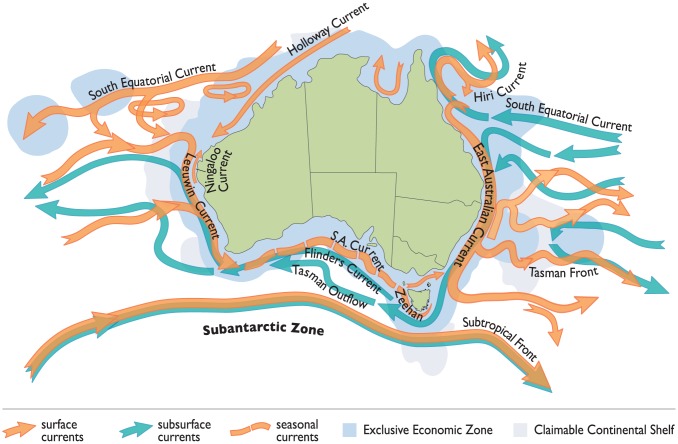
The major oceanic currents in the Australian region. Adapted from [13].

Australia is the only continent with two poleward-flowing boundary currents transporting tropical waters along both the east and west coasts (Fig. 2). The East Australian Current (EAC) carries water south from the tropical Coral Sea along the east coast of Australia into the Tasman Sea, dominating waters between 18° S and 35° S. As the EAC moves south, it separates from the coast between 30.7 S and 32.4 S around 50% of the time; however, it can separate anywhere between 28 S and 38 S, with upper layers flowing eastwards to form the Tasman Front and deeper layers spawning warm-core eddies that continue to move south as the EAC extension [23], [24]. The Leeuwin Current originates from the Timor Through Flow, flowing south from the North West Cape of Western Australia, and then east along the continents southern coast for a distance of up to 5500 km [25]. Both current systems are seasonally influenced, with the EAC extending further into southern waters along the eastern Tasmanian coast in summer [23] and the Leeuwin Current extending further eastwards to flow across the Great Australian Bight (GAB) and then southward along the west coast of Tasmania in winter [25].

#### Phytoplankton provinces

There are strong latitudinal gradients in phytoplankton around Australia with tropical communities dominated by small cyanobacteria such as *Prochlorococcus* and *Synechococcus* with increasing abundances of nano and microplankton further south [26]. Australia has six phytoplankton provinces based on dinoflagellate associations with specific water masses and other marine microalgal species communities that are repeatedly found in certain locations [27]. These include: 1) the diatom dominated shelf waters of north-west Australia [28], the Gulf of Carpentaria [29], Arafura and Timor Seas; 2) tropical oceanic waters with a predominately dinoflagellate community including *Histioneis* and *Ornithocercus* [30], [31], with these neritic communities containing tropical diatoms, cyanobacteria and dinoflagellates carried southwards by the Leeuwin Current and EAC; 3) the fast-growing nanoplankton diatom dominated shallow waters of the Great Barrier Reef lagoon [32]; 4) the productive temperate neritic coastal waters of New South Wales, Tasmania, Victoria and South Australia with a stronger seasonal progression from diatoms to dinoflagellates such as *Ceratium* and *Dinophysis* and *Noctiluca* [33]; 5) a highly variable oceanic transition zone which sits in-between the temperate and tropical phytoplankton communities [34] and; 6) a sub-Antarctic phytoplankton province along the sub tropical front that supports a significant coccolithophorid bloom in summer [35] dominated by *Emiliania huxleyi* in this colder and deeply mixed water mass [36].

#### Australia's network of Marine Reserves

In 1998, the Commonwealth and state and territory governments committed to the creation of a National Representative System of Marine Protected Areas. Six interconnected marine regions around the continent have built upon existing state and federal plans, such as the Great Barrier Reef Marine Park (GBRMP), resulting in a comprehensive network of proposed marine reserves [37]. Within three nautical miles of the coast these reserves are generally state controlled, with the exception of some coastal regions in the GBRMP. Reserves situated beyond 3 nm from the coast out to the 200 nm exclusive economic zone are predominately controlled by the Commonwealth government. Sustained observing has been identified as critical for ongoing planning, research and monitoring within the management plans of the proposed marine reserve networks [37].

#### Regions of priority for the IMOS nodes

The science plans for each of the six IMOS nodes identified a number of key research themes (available at www.imos.org.au/nodes). Research conducted under the blue water and climate observing node is primarily focused on oceanography at continental to global scales, and in particular, on climate forcing and variability, multi-decadal change and linkages between offshore and coastal processes. Research conducted under each of the regional nodes investigates the relationships between physical, chemical and biological processes at Australian state or multi-state scales and between local oceanographic processes and regional climate phenomena such as the El Niño–Southern Oscillation (ENSO). These include describing the EAC and Leeuwin Current and their eddy fields (Fig. 2), continental shelf processes and biological responses to these systems, as well as the highly tidally influenced coastal waters of Australia's north. For instance, the complex coastal oceanography of the south-east region has been identified as an area of significantly warming ocean temperatures [38] and unprecedented warm sea surface temperature anomalies on the west coast of Australia, the Ningaloo Niño, has also recently been described [39]. Characterizing and understanding the oceanography and ecosystems within these regions are of high priority for the regional nodes.

#### Additional NRS

Based on the above criteria, Australia's coastal waters can be subdivided into five distinct environmental regions: 1) The tropical north, characterized by broad, shallow seas, strong tidal influences, high sediment loading and influenced by the Holloway Current; 2) The Great Barrier Reef (GBR) lagoon, which is influenced by the EAC and Hiri Current; 3) The south-east, extending from the sub-tropics to the cool temperate waters of Tasmania, characterized by a narrow shelf dominated by the EAC; 4) The central south including the broader shelf areas of the GAB and influenced by the Leeuwin and Flinders Currents and periodic upwelling; and 5) the south-west characterized by a narrow continental shelf dominated by the Leeuwin Current (Fig. 1 and 2).

Each of the five areas were identified as requiring at least one NRS and multiple NRS sites if required by scale to ensure that all major current systems, each phytoplankton province, marine reserve network region and IMOS regional coastal node were able to be sampled. A further six sites were identified to complement the existing sites, thus creating the NRS network. These included Darwin in the tropical north, Yongala in the GBR lagoon, North Stradbroke Island in the sub-tropical east, Kangaroo Island in the central south, Esperance in the south-west and Ningaloo Reef in the north-west (Table 1, Fig. 1). The oceanographic features of each of these sites are described in supplementary material.

**Table 1:** Features of the nine National Reference Stations.

	Yongala	Nth Stradbroke Is	Pt Hacking	Maria Is	Kangaroo Is	Esperance	Rottnest Is	Ningaloo	Darwin
Year deployed	2007	2011	2009	2008	2008	2008	2008	2010	2009
Latitude (°S)	19°18.5	27°20.5	34°05.0	42°35.8	35°49.9	33°56.0	32°00.0	21°52.0	12°24.0
Longitude (°E)	147°37.1	153°33.73	151°15.0	148°14.0	136°26.8	121°51.0	115°25.0	113°56.82	130°46.1
IMOS node	QLD	QLD	NSW	TAS	SA	WA	WA	WA	WA
Reserve network	GBRMP	TE	TE	SE	SW	SW	SW	NW	N
Plankton province	GBR	GBR	TN	TN	TN	TO	TO	TS	TS
Depth (m)	27	63	100	90	110	50	50	55	20
Operator	AIMS	CSIRO	SIMS	CSIRO	SARDI	CSIRO	CSIRO	AIMS^a^	AIMS
No. services (per yr)	2	3	6	3	3	3	3	2	2
No. samples (per yr)	12	12	12	12	8	4	12	4	4
Telemetry	Yes	Yes	No	Yes	No	No	No	No	Yes
Shelf processes	C,M	C,E,U	C,E	CZ	C,U	C,U	C,E,U	C,U	C,M
Currents	EAC	EAC	EAC	EAC,ZC,LC	LC,FC,LU,U	LC	LC	LC,NC	HC

a Water samples at Ningaloo are collected by CSIRO and AIMS. IMOS Node: QLD: Queensland; NSW: New South Wales; TAS: Tasmania; SA: South Australia; WA: Western Australia. Marine Reserve Network: GBRMP: Great Barrier Reef Marine Park; TE: Temperate East; SE: South-East; SW: South-West; NW: North-West; N; North. Phytoplankton provinces: GBR: Great Barrier Reef; TN: tropical neritic; TO: tropical oceanic; TS: tropical shelf. Institutes: AIMS: Australian Institute of Marine Science; CSIRO: Commonwealth Scientific and Industrial Research Organisation; SARDI: South Australian Research and Development Institute; SIMS: Sydney Institute of Marine Science. Shelf processes: C: currents; CZ: connection zone; E: eddies; M: monsoonal influences; T: tidal influences; U: upwelling. Current systems: EAC: East Australian Current; FC: Flinders Current; HC: Holloway Current; LC: Leeuwin Current; LUC: Leeuwin Undercurrent; NC: Ningaloo Current; ZC: Zeehan Current.

### The sampling program

Water and plankton sampling were conducted under permit # QS2010/MAN111a

(Marine Parks Act 2004) and under permit #95170 (Fisheries Act 1994). Other field work permits were obtained from: CSIRO Oceans and Atmosphere, GPO Box 1538, Hobart, Tasmania 7001, Australia, CSIRO Oceans and Atmosphere, 41 Boggo Rd, Dutton Park, Queensland, 4102, Australia Centre for Applications in Natural Resource Mathematics, University of Queensland, St. Lucia, Queensland 4072, Australia Australian Institute of Marine Science, PMB #3 Townsville MC, Queensland 4810, Australia School of Mathematics, University of New South Wales, Sydney, New South Wales 2052, Australia South Australian Research and Development Institute, PO Box 120 Henley Beach, South Australia 5022, Australia.

The sampling program for the NRS was designed on five requirements: 1) expansion of the predominately physical dataset of the historical sites to an integrated suite of physical, chemical and biological oceanographic parameters; 2) an increased rate of sampling to allow resolution of daily cycles, capture of rare events and improved statistical power to detect change over time; 3) continuous deployment of *in-situ* sensors, which are not affected by adverse weather that prevents field work; 4) collection of species level information for both phytoplankton and zooplankton to determine ecosystem response to environmental change; and 5) cross validation of data derived from sensors and water sampling for quality control.

Continuous and robust data collections for many properties of the water column are now possible via *in-situ* sensor systems. Some chemical parameters and species-level data, however, are unable to be collected robustly via such systems. As a result a sampling program consisting of moored and serviced sensor packages coupled with regular vessel-based sampling and laboratory analysis was instigated.

#### Moored sensors

**Figure 3:**
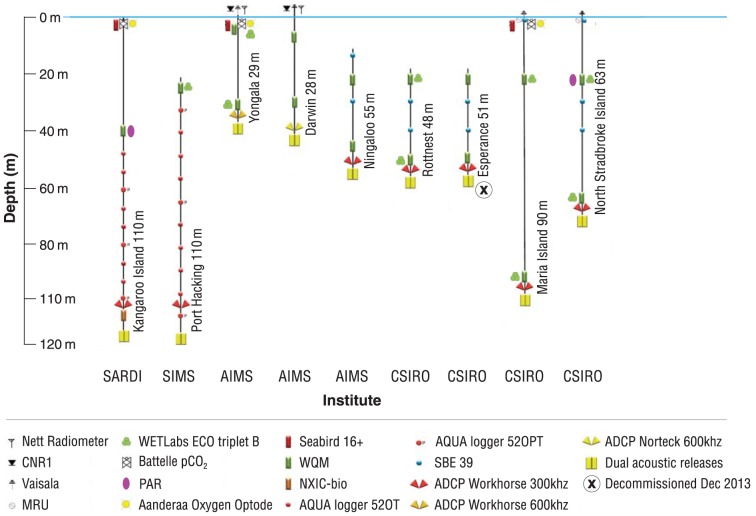
Schematic representations of the moored sensor design for each of the National Reference Stations. Note, not all instruments are deployed onto single mooring lines.

The *in-situ* moored sensors of the NRS network comprise a number of instruments deployed at all sites that measure a set of core parameters. Additional sensors, identified by the science nodes to collect data for addressing specific science questions, are deployed at a subset of sites (Fig. 3). Roll out of the network has been phased, resulting in variation in commencement dates (Table 1).

#### Core parameters

The high temporal resolution data (Table S1 in S2 File) collected at each NRS site are mostly generated by paired - shallow and deep deployed - Water Quality Monitors (WQM; Wetlabs, Philomath, USA) and a bottom mounted Acoustic Doppler Current Profiler (ADCP; Teledyne RDI, Poway, USA). Each WQM consists of a pumped conductivity, temperature and depth (CTD) sampler (SBE MicroCAT, Seabird, Seattle, WA, USA), oxygen sensor (SBE 43, Seabird, Seattle, WA, USA) and a combined flourometer and turbidity sensor (FLNTU; EcoPuk, Wetlabs, Philomath, USA). The fluorescence excitation wavelength of the EcoPuk in the WQM is 470 nm while the emission wavelength is 695 nm. For the turbidity measure the EcoPuk's wavelength is 700 nm. A variation in instrumentation occurs at Kangaroo Island, where the near surface sensor package consists of a NXIC CTD (Falmouth Scientific, Cataumet, MA, USA) with an Optode oxygen sensor (Aanderraa, Bergen, Norway) and an EcoPuk FLNTU. A Photosynthetically Active Radiation (PAR) sensor (QSP-2100, Biospherical, San Diego, USA) is also mounted on the Kangaroo Island mooring near the FLNTU at 40 m depth (Fig. 3).

Water column current velocity and direction are measured using bottom-mounted and upward-looking 300 KHz ADCPs at all sites, except Yongala and Darwin, where 600 KHz ADCPs are deployed (Table S1 in S2 File). These higher frequency ADCPs are better suited to resolving the shallower water and fast tidal patterns of these two sites and are used to infer multi-directional internal waves.

ADCPs are deployed either as separate sub-surface moorings (NRS ROT, NIN, ESP, KAI, PH) or landers (MAI, YON, DAR, NSI) to avoid interactions with mooring lines. Optical sensors are placed in-line with the mooring wire, as they are designed to take point samples; though the actual sampling volume of the EcoPuks is not characterized by the manufacture. For the FL signal, interactions with the mooring wire or cage will only occur with fouling as the sensor registers fluorescence. The NTU signal, however, has a greater possibility of wire interactions. We analyzed this data for MAR stations and found only a small number of points, less than 1%, showed any possible interaction with the wire (data available on-line).

The frequency of sampling by the sensors is standardized across the network. Data is collected from sub-surface sensors at one-second intervals across one minute of continual burst sampling every 15 minutes. Data are logged internally and data delivery is delayed until moorings are serviced. The moorings servicing interval seeks to maintain high quality data sets. Local conditions (e.g. degree of bio-fouling) determine the servicing periods, which vary from monthly at the Port Hacking site to six monthly at Yongala (Table 1). At those sites with telemetry capabilities (see next section) a value is calculated for parameters across each burst to provide data averages in near real time.

#### Additional sensors and telemetry

Meteorological data are available for all NRS sites; weather stations (WXT520, Vaisala, Helsinki, Finland) capable of measuring barometric pressure, wind speed, wind direction, air temperature, liquid precipitation and relative humidity along with a surface temperature sensor (SBE 39, Seabird, Seattle, WA, USA) have been deployed in a surface float sensor package at Maria Island, Darwin, Yongala and North Stradbroke Island (Table S2 in S2 File). Complimentary meteorology data are available at the Port Hacking, Kangaroo Island, Esperance and Rottnest Island sites via Australian Bureau of Meteorology stations located in close proximity to the NRS sites, or in the case of the Ningaloo station, a nearby automatic weather station maintained by the Australian Institute of Marine Science (AIMS). Motion reference units (3DMGE1, Microstrain, Williston, USA) capable of recording wave height are also deployed at Maria Island and North Stradbroke Island. Samples from the weather station and MRU sensors are telemetered and comprise 5 minutes of data acquisition each hour, which is then summarized into averages. Individual samples are taken at rates of 5 Hz for the MRU sensors and then Fourier transformed into a data package of acceleration magnitudes.

The surface float electronic packages deployed at Maria Island, Darwin, Yongala and North Stradbroke Island also allow for telemetry of near real-time data for SST, along with sub-surface sensor data from the WQMs on an hourly basis. Transmission of data was originally via satellite telephone, but more recently this has been transferred to a system that first attempts to send data via mobile phone before defaulting to the satellite system for those stations, (Maria Island, North Stradbroke Island and Darwin) which are within range of mobile telephone cell towers.

The NRS also provides capability for the monitoring of carbon dioxide and ocean acidification [40]. Sensors for measuring CO_2_ (Battelle pCO_2_, Battelle, Columbus, OH, USA), oxygen (Oxygen Optode, Aanderaa, Bergen, Norway) and salinity and temperature (SBE 16 +, Seabird, Seattle, WA, USA) have been deployed at the Maria Island, Kangaroo Island and Yongala stations (Table S3 in S2 File), with data telemetered via an independent rudics system.

Many stations have other, additional sensors which log data to be provided in a delayed mode. Stations at Maria Island, Rottnest Island, Port Hacking and North Stradbroke Island have an additional FLNTU (Ecotriplet B, Wetlabs Philomath, USA), which were first deployed between 2012 and 2013, and are capable of measuring blue, green and coloured dissolved organic matter (CDOM) wavelengths (Fig. 3, Table S3 in S2 File). These are co-located and complement the wavelength of the standard EcoPuk FLNTU on the WQMs. For the Ecotriplet's two scattering band wavelengths, blue was set at 470 nm, while green at 532 nm. For the CDOM florescence the excitation wavelength was set at 370 nm and emission at 470 nm. Another Ecotriplet B is planned to be deployed at the Yongala NRS, as are PAR sensors for the Maria Island station. Combined bio-optical observations from these sites will be used for observing particle and phytoplankton dynamics, modelling of the underwater light climate for primary production, and identifying proxies linking optical observations to biogeochemical properties. Additional salinity and temperature sensors have been placed on the Kangaroo Island, Port Hacking, Ningaloo, Rottnest Island, Esperance and North Stradbroke Island stations to better resolve local physical water column properties (Fig. 3, Table S3 in S2 File).

#### Vessel-based sampling

The long-term water sampling program, established at the historical sampling sites, has been expanded into a more comprehensive program (Table S4 in S2 File). The program generally replicates the monthly time scale of the historic sampling frequencies at Port Hacking, Maria and Rottnest Islands, as this has proved to be sufficient to detect multi-decadal change [18]. However, local conditions and logistical constraints required reduced sampling at some sites. Due to the extreme nature of the tides at the Darwin station, sampling was reduced to four times a year but spread across the tidal cycle. This quarterly sampling was designed to capture the variability in sediment and nutrient loads across dry and wet seasons and spring and neap tides. Samples at Kangaroo Island are collected eight times a year due to limited availability of research vessels, and four sampling times occurred at Ningaloo and Esperance to coincide with remote field trips to service the moorings and the seasons.

Detailed documentation of the vessel-based sampling methods are provided in an NRS Biogeochemical operations handbook and NRS standardized profiling CTD cast procedures which are published on the IMOS website (imos.org.au/anmndocuments). Briefly, sampling comprises: 1) vertical profiling sensor measurements of conductivity (salinity), temperature and depth, oxygen, fluorometry and turbidity; 2) Niskin bottle samples at discrete 10 m intervals for measurements of total dissolved inorganic carbon, alkalinity and nutrients; 3) a combined water column sample from all Niskin bottles for phytoplankton and pigments; 4) zooplankton samples from a plankton drop net; 5) and additional Niskin bottle water samples taken adjacent to moored instruments to allow for cross validation and characterization of data collected by the moored sensors; and 6) measurement of turbidity with a Secchi disk.

**Figure 4:**
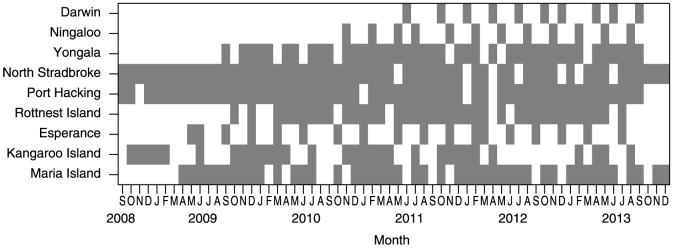
Numbers and timings of biogeochemical water and plankton samples taken on site at all stations from the interception of the expanded sampling program.

Sampling at each of the sites is conducted by staff from the nearest partner institute. Numbers of samples planned to be taken at each of the nine sites are provides in Table 1. Actual biogeochemical water and plankton samples taken, which includes gaps and timings, from the interception of the expanded sampling program to submission of the manuscript are provided in Fig. 4.

#### Laboratory analysis

Samples of similar suites of parameters are freighted to centralized laboratories for analysis by specialized teams of technicians and scientists. Three different laboratories at CSIRO's Oceans and Atmosphere (OA) Flagship in Tasmania conduct analysis on hydrochemistry, plankton pigments and carbon respectively. Tropical and temperate phytoplankton and zooplankton samples are processed at the OA laboratories in Queensland, while cool temperate phytoplankton samples are processed at the Australian Antarctic Division in Tasmania. Pico-plankton analysis is undertaken by the Australian Microscopy and Microanalysis Research Facility at the University of Western Australia.

### Results

The expanded NRS system has increased the extent of sampling: extending stations into the tropics, the number of parameters measured from ten to more than 50 and, through placement of moored sensors, increased the frequency of sampling of some parameters by up to five orders of magnitude. Temperature and salinity, which were historically measured using manual methods at a maximum of three times per season are now measured by *in-situ* sensors up to 480,000 times per season. Data collected now includes not only physical and chemical parameters, but also biological indicators at two trophic levels.

### Applications

**Figure 5:**
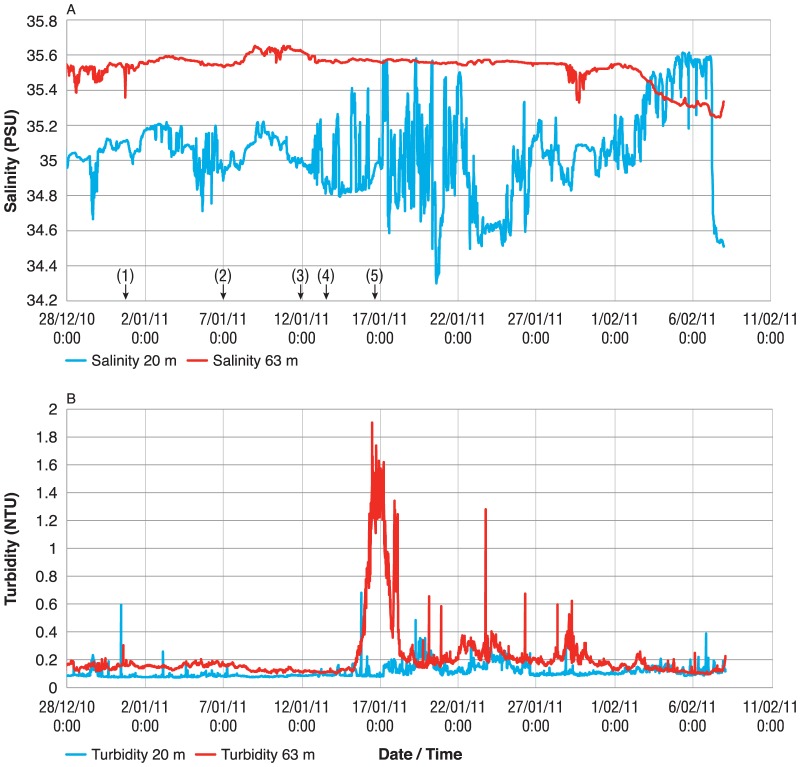
Time-series of salinity (A) and turbidity (B) measurements collected at the North Stradbroke Island station during a flood. The event was related to a controlled release of water from a large dam following rain across an already saturated catchment, which commenced on the 1^st^ January 2011 (1), and continued to the10^th^. Peak rainfall occurred on the 7^th^ (2), and water released from the dam late on the 11^th^ (23:30) (3). Major flooding occurred across Brisbane city from the 12–14^th^ (4), and peak turbidity of the plume disgorging to NRS NSI, which is ∼ 41km distant from the river mouth, was observed on the 16^th^ (5).

Measurements provided by the moored sensors of the NRS allows for continuous monitoring of episodic events, which are common in coastal oceanography [1]. As an example, salinity and turbidity sensors at the North Stradbroke Island NRS were deployed during large scale flooding events in 2010-11 [41], [42]. As freshwater carrying flood debris and sediments moved out of coastal catchments, the stations 20 m CTD sensor detected an increased variability in surface salinity (Fig. 5a) while the sensor deployed near the sea floor detected an increase in turbidity (Fig. 5b). Due to the weather conditions across this period, it would have been unsafe to have collected data from a small boat and monthly sampling would also not have captured the variability of the salinity changes and turbidity plumes across scales of hours, days to weeks.

**Figure 6:**
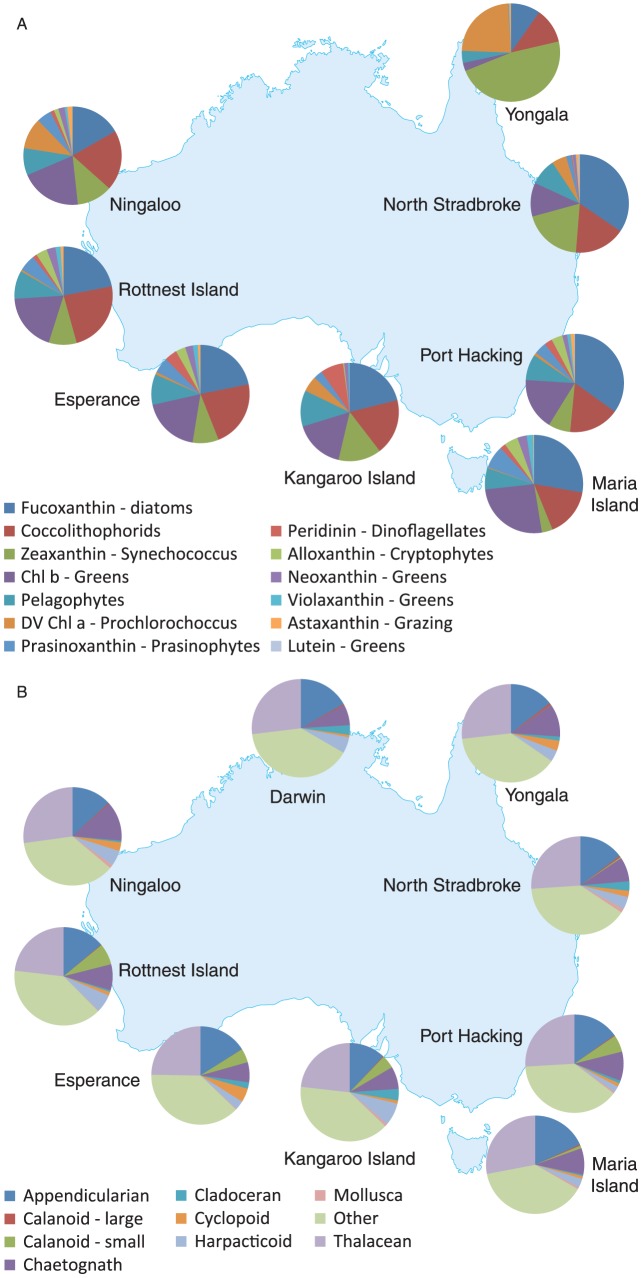
Average phytoplankton pigments normalized to chlorophyll a at all national reference stations sampled between February 2009 and December 2010 (n  =  190) (A). Major functional groups of zooplankton sampled with a 100 µm drop net across the NRS network (B).

The NRS network has, for the first time, provided descriptions of the relative concentrations of phytoplankton pigment types (Fig. 6a) and zooplankton species (Fig. 6b) across all of the currently defined phytoplankton provinces, with the exception of the sub-Antarctic province, identified around Australia. While connectivity is reflected in the pigments (Fig. 6a), cell counts and analysis by flow cytometry, there is also considerable evidence of strong latitudinal gradients. For example, down the east coast of Australia from the NRS at Yongala (∼19°S and 148°E) where zeaxanthin was 52% of total chlorophyll *a* to just 3% at Maria Island some 24°S further south (∼43°S and 148°E). This trend is consistent with, albeit somewhat faster than, the 1.3% decrease in all picoplankton abundance per degree of latitude previously reported [33]. In the tropics phytoplankton tend to be dominated by di-vinyl chlorophyll *a* and zeaxanthin, markers for the picoplanktors *Prochlorococcus* and *Synechococcus*, respectively.

For the zooplankton, the Copepods (calanoids, cycopoids and harpacticoids) dominate the community, constituting 45–70% of the observed abundance across the network (Fig. 6b). The southernmost NRS of Maria Island, which is located in the relatively cool and productive Tasman Sea, has the greatest proportion of copepods. Cladocerans are generally a coastal group and are found in high numbers in waters of southern Australia, including Port Hacking, Rottnest and Kangaroo Island NRS. Mollusc larvae were found at the Ningaloo and Yongala NRS, which are both adjacent to coral barrier reefs. Appendicularians, which feed on bacteria and picophytoplankton, are most common (∼15%) along the east coast, less common on the south coast, and absent or rare on the west coast.

**Figure 7:**
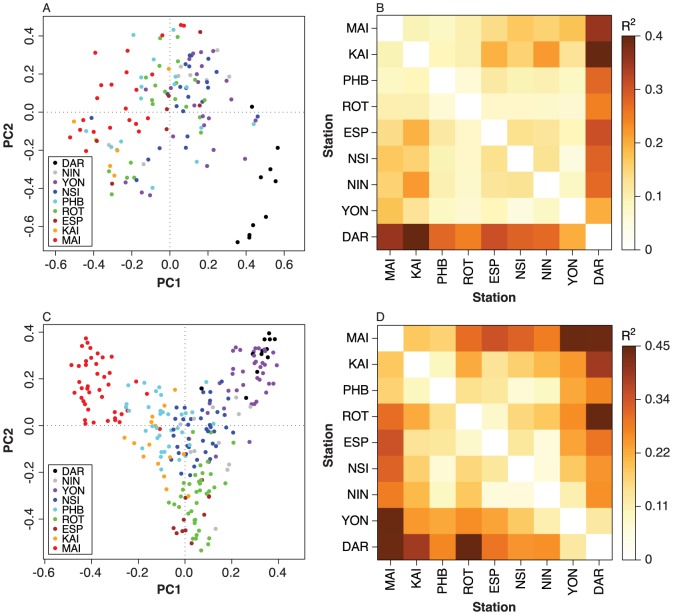
PCA sample scores (A, C) and heatmaps of R^2^ values from pairwise pMANOVA tests (B, D) for phytoplankton (A, B) and copepods (C, D). In the PCA plots, samples are coloured by station. The R^2^ values in the heatmaps provide a measure of how distinctly the samples from each station are separated from those of each other station. For clarity, the stations in the heatmaps have been sorted by the median sample scores from RDAs constrained by station.

Principle component analysis (PCA) and permutational analysis of variance (pMANOVA) for phytoplankton taxa as well the copepods (Fig. 7) also demonstrates how these communities separate over the continental scale of sampling. The final community matrices included 136 samples and 291 taxa for phytoplankton and 244 samples and 183 species for copepods. The community matrices were transformed with the fourth-root transformation and then with the Hellinger transformation [43]. Variation in species composition was summarised using transformation-based PCA (Legendre 2012). Differences among stations were examined with pMANOVA. Variation between pairs of stations was examined with pairwise pMANOVA. Pairwise *p* values were adjusted for multiple comparisons using the Holm method and visual inspection of pairwise RDAs was also performed to check that the results were reasonable. We note that our pMANOVA tests were unbalanced with respect to station, year and season, hence the individual pairwise tests will be less robust than general trends. Analyses were performed with an R package [44].

For phytoplankton, the first two PCA axes accounted for 12% and 6% of the variance in species composition respectively. The first axis tended to order stations from south to north (Fig. 7a) and is in reasonable agreement with our understanding of how the plankton community is structured [27]. There is a general north-south gradient on the first axis with large overlaps between the temperate stations (Fig. 7a). This most likely reflects the connectivity between these provinces by the major pole ward flowing current systems. Overall, station explained a significant proportion of the variation in species composition (*R* ^2^  =  24%, *p* <0.0001). Pairwise pMANOVA tests indicated that the clearest separations were between the most northern and southern stations (Fig. 7b). Darwin stands out as being clearly separated from all other stations (Fig. 7b). All pairs of stations were significantly different (adjusted *p* values are all less than 0.05).

For copepods, the first two PCA axes accounted for 14% and 7% of the variance in species composition respectively. The first axis tended to order stations from south to north and the second axis separated the south-western stations (Rottnest and Esperance) from the other temperate and subtropical stations (Fig. 7c). So we have separation in community structure between the cool temperate waters in the South at the Maria Island NRS and all other stations, as well as longitudinal division between the western stations at Rottnest and Esperance and at the tropical stations of Darwin and Yongala NRS, with the tropical north-west Ningaloo NRS bridging these stations (Fig. 7). There was also a central group of stations in the PCA comprising the North Stradbroke Island, Port Hacking and Kangaroo Island NRS, which are located in the South and East temperate and subtropical zones. Overall, station explained a significant proportion of the variation in species composition (*R* ^2^  =  37%, *p* <0.0001). Pairwise pMANOVA tests indicated that the clearest separations were between the most northern and southern stations (Fig. 7d). Even more so than phytoplankton all pairs of stations were highly significantly different (adjusted *p* values all less than 0.01).

**Figure 8:**
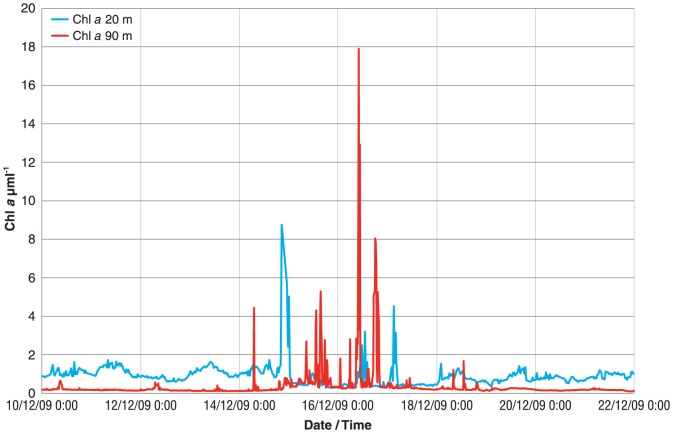
Time series of chlorophyll measured by the combined flourometer and turbidity sensor deployed at the Maria Island station during a December 2009 bloom event.

In recent years, the research community has benefitted from increased availability of observations from bio-optical sensors of variables with both biogeochemical and ecosystem relevance. These include fluorescence proxies for Chlorophyll *a*, and optical backscattering or attenuation coefficients, which are used as proxies for particulate organic carbon and coloured dissolved organic matter (CDOM) [45]. By providing continuous FLNTU measurements at two depths at each of the NRS sites, examples of rare, episodic and short lived phenomenon, such as algal blooms within the water column can now be made (Fig. 8). Determining if these are real events or artifacts of bio-fouling is assisted by the continuous deployment of dual depth stratified sensors. Actual blooms are discreet and short lived, while fouling artifacts are continuous till servicing of the mooring. Fouling also tends to pre-dominantly occur at the shallow (20 m) rather than deep (90 m) sensor. Hence, detection of peaks in the Chlorophyll *a* proxy at both sensors, which is facilitated by seasonal full water column mixing at the Maria NRS site, provides another piece of evidence that this is an actual bloom.

**Figure 9:**
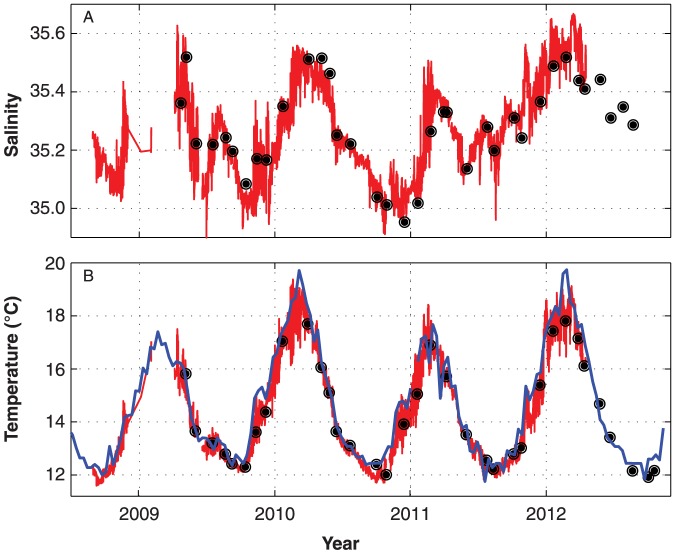
Quality control comparison of (A) salinity at 20 m, measured by the moored CTD sensor (continuous red line) and the monthly bottle sampling program (closed black circles) and (B) water temperature at 20 m measured by the moored CTD sensor (continuous red line), the monthly sampling program (closed black circles), and from a gridded and interpolated satellite sea surface temperature product (blue line) at the Maria Island station.

As the historical data collection methods of monthly sampling have continued as part of the expanded program, there is opportunities to cross validate *in-situ* sensor data with this independently collected point data, for quality control purposes. For example, at the Maria Island NRS, salinity and temperature at 20 m measured by the moored CTD sensor compared well with monthly bottle sampling and CTD cast data (Fig. 9a). The 20 m temperature signal also compares well to a multi-day composite gridded and interpolated satellite sea temperature product [46] (Fig. 9b). The Maria Island NRS is approximately 7.5km off shore from Maria Island which is itself separated by 4 km from the mainland of Tasmania. The satellite temperature product was based on a 4 km resolution with the closest pixel to the Maria NRS used for comparison; so there is a small risk of contamination by the pixels proximity to land. However, the satellite plot showed good agreement with the two *in-situ* measures and thus provides information on both seasonal and inter annual variation, including multi-degree differences in summer temperatures between the 2009 and 2010 years.

### Challenges and solutions

As might be expected with any continental-scale monitoring program, numerous operational challenges were faced during development.

#### Instruments and sampling

Time series of oceanographic parameters at the historical stations was, in the past, solely based on monthly small boat sampling excursions. This sampling rate however, was termed ‘pseudo-monthly’ [38], as many months went un-sampled due to bad weather. As can be seen from the continued and expanded biogeochemical sampling (Fig. 4) this issue has continued. With the deployment of *in-situ* instruments at the sites, we have ameliorated this sampling frequency problem somewhat, allowing for monitoring to continue even in inclement conditions (e.g. Fig. 5). However, this approach posed new challenges. The scales across which sensors sampled the environment in some cases had to be adjusted (e.g for high turbidity at the Darwin NRS) to ensure that sampling was adequate for individual site conditions. Often instruments were either beta or early production run versions, which required on-going consultations with the manufactures to ensure reliable data at relevant measurement scales could be obtained.

Continuous collection of data throughout the year requires a pool of instruments to swap into and out of the moorings. This required the implementation of standard calibration methods for sensors like salinity and temperature, and development of new calibration procedures for other sensors like FLNTU to ensure precise and accuracy of the time series. To achieve this each institute involved in the NRS supports a National Association of Testing Authorities (NATA) certified calibration laboratory, which is operated by the CSIRO. To test that calibration co-efficients have been correctly applied, concatenation of all mooring deployment datasets into continuous time-series is also required. In particular this allows for the checking for step changes in data between individual sensors.

#### Data processing and Quality Control

For many of the parameters collected by the NRS, quality control processes and data standards did not exist prior to the start of the project. For example, basic broad scale identification guides for plankton were lacking. Sampling at the NRS for plankton is now being used to develop national data standards. Unlike samples taken by other methods, such as the continuous plankton recorder, zooplankton samples from the NRS are usually undamaged and are being used as reference material for the online atlas guide for Australian zooplankton [47]. Another guide, for Australian tropical phytoplankton, is also in preparation using material collected from the NRS (Gustaaf Hallegraeff pers. comms).

To maintain data standards for sampling, analysis and preparation and freight of samples to the centralized processing laboratories, regular meetings and training is coordinated from the CSIRO's laboratories in Hobart Tasmania. Each year talks and workshops are also held in Hobart as part of the annual QC summit to table issues with instruments and sampling and to develop solutions. The agendas, presentations and summary reports from the Summits are loaded onto the IMOS website (imos.org.au/qc2013).

It soon became apparent that numerous QC standards and approaches are available across the global community and that these needed to be synthesized into an appropriate framework [48]. The program has invested substantial time and effort into developing methodological tools for integrated biogeochemical sampling systems, methods for extracting and processing profiling sensor data and general data quality control standards for high frequency data (imos.org.au/facility_manuals). As part of the broader ANMN facility a Matlab Toolbox (code.google.com/p/imos-toolbox) was developed in conjunction with eMII that allows for parsing of data from a wide variety of instruments and basic quality control of data streams.

#### Data management

Data from the NRS are uploaded to the eMII facility and made available to the public through the Australian Ocean Data Network (AODN) (portal imos.aodn.org.au/webportal). Sensor data are stored using the network Common Data Form (netCDF) system, a set of software libraries and machine-independent data formats that support the creation, access, and sharing of array-oriented scientific data. The Matlab toolbox is used to parse data into netCDF format with metadata added from a deployment database. The toolbox is maintained and version controlled by eMII, which distributed up-dates to the IMOS community as they become available.

Historic data (1941 – 2008) are stored as.csv files, as are data from the water sampling program. Though for ease of access, these have been consolidated into an Oracle database which allows for data harvesting of multiple data types simultaneously and concatenation of monthly sampling.

While a central data co-ordinator was useful during initial phases of the networks establishment this proved problematic when large amounts of data starting moving through the systems. Having all data go through a centralized point, meant reasons for delays in data availability where obscured from the eMII facility, which serves the data to the public. It was unknown whether the issue was the clearing house, the samplers, laboratories or mooring service teams. We solved this by moving the data upload processes to a distributed approach with each team now independently transferring data directly to eMII. Delays are therefore constrained to individual data streams or sites and are easily identifiable. Close liaison between the ANMN Facility Leader and eMII has been critical to resolve the numerous data workflow issues across the NRS.

#### Design, governance and resourcing

Given the broad nature of the community of researchers that were involved in the establishment of the network and who are served by the data, some tension in the design and approaches for data integration were inevitable. An NRS Scientific Steering Committee comprised of researchers involved in the program was tasked with the development of a rationale, design and implementation plan [49] specifically aimed at detailing and resolving any design issues. In addition, as part of the mooring network, yearly business plans and quarterly milestone reports for the NRS have also been implemented. A variety of strategies have been developed to maintain ongoing and close relationships between staff from the multiple agencies involved in the NRS. These include regular meetings of network developers, users and managers via a yearly business planning meeting as well as the separate annual QC summit. These formal gathers draws together both leaders and practitioners to identify problems and develop solutions. The facility leader also provides a central point of leadership, and is based in close proximity to the eMII data centre and the IMOS office.

As part of the NRS rationale and implementation plan [49] it was estimated that the network cost approximately $1.9M per annum to operate, excluding capital depreciation. Ongoing consideration of design and resourcing of the NRS network indicated that quarterly BGC sampling at the most remote NRS sites, Ningaloo and Esperance, did not provide sufficient scientific value, relative to the cost involved. Monthly BGC sampling was never affordable at these two remote sites. The lower rate of sampling (i.e. quarterly) was instigated during establishment of the NRS network, for cross validation of sensor data, sensor characterization to local conditions and development of data standards such as national guides for plankton identification. However quarterly BGC sampling ceased at these two sites in 2013 (Fig. 4), and the mooring at the most remote site (Esperance) was decommissioned in December 2013 (Fig. 3).

## Discussion

The combination of moored instruments and repeated biogeochemical sampling at known locations are the only viable strategy available, at the moment, for developing spatially-explicit time series in relatively shallow continental shelf waters. It is only through development of long-term datasets that trends such as the coastal response to global climate change or other anthropogenic effects can be separated from natural variability [50]. By combining *in-situ* sensors with more traditional sampling methods, the NRS network is beginning to address some of the gaps in biological oceanographic observations identified by the global research community, such as continuous measurements of optical proxies for abundance of phytoplankton as well as observations of community structures for both phytoplankton and zooplankton [10]. The reference nature of the stations also means they are long term sentinels that provide the context to climate driven changes to biological communities, such as poleward shifts in critical sea temperatures for larval mortality of invasive sea urchins [51]. The geographic spread of the stations has also allowed them to play a broader role in helping to track anomalous warming events from the North West to the South East of the Australian continent [19], [38], [39].

Besides an increase in utility, by continuous sampling from the moored sensors, the new NRS *in-situ* sensor array allow shorter term phenomenon to be characterized. This include tides, eddies, stratification of the water column, blooms of phytoplankton and associated changes to O_2_ and CO_2_ from photosynthesis and respiration. Also, daily cycles are now able to be observed as observations continue into the night, as are unusual or stochastic events. A dramatic example of this was the timeline of variability in salinity and turbidity observed in January 2011 at the North Stradbroke Island NRS (Fig. 5). This corresponded directly with the 13^th^ January floods of the adjacent Brisbane River which resulted in extensive flooding of the city of Brisbane [41]. While these floods occurred within the context of a strong La Niña event, which resulted in a wide scale precipitation abnormality across Australia [42], this particular pulse of water is considered a “dam release flood” caused by a controlled release of water from a major structure in the catchment, the Wivenhoe Dam [41].

The increased spatial and temporal frame and the greater number of parameters sampled by the NRS that now includes biological sampling have expanded the scope of the program. It is now similar to a number of other long term monitoring programs, such as the Continuous Plankton Recorder survey, which has monitored plankton on a monthly basis in the North Atlantic since 1946, and has shown strong biogeographical shifts in all copepod assemblages, which are related to both the increasing trend in Northern Hemisphere temperature and the North Atlantic Oscillation [52]. Another similar multi-disciplinary program has focused on the Western English Channel from 1888 to the present [53]. This oceanographic and ecological research program is similar to the NRS in parameter scope but has involved more intense sampled over smaller spatial scales (i.e. English Channel vs. Australian continent). Like the historic NRS sites the observations span significant periods of warming (1921–1961; 1985– present) and cooling (1962–1980), however data collection has included more trophic levels such as the inter-tidal zone and demersal fish. Changes observed over this period in zooplankton, pelagic fish, and larval fish and the collapse of an important herring fishery all appear to have climate as a forcing factor. These long-term data also yield important insights of anthropogenic disturbances such as fisheries exploitation and pollution and provided advances in diverse scientific disciplines either generated from research or that undertaken alongside the long-term data series.

Both of these studies demonstrated the potential long term benefits of our continental scale NRS network for tracking the spatial and temporal variation in physical events and the flow-on consequences to biological systems. Though many of these British studies were terminated during organizational restructures in 1987–1988, there has since been a resurgence and expansion of sampling due to an increase in interest in long-term environmental change. Like these international sampling programs, we share common challenges in maintaining continuous data streams across extended sampling, with the seemingly inevitable gaps appearing in our databases (Fig. 4).

At a local or regional scale, coastal observing systems such as the Chesapeake Bay Interpretive Buoy System (CBIBS) [54], provides a sister program to the NRS, utilizing the same sensor technology. Like the CBIBS our *in-situ* sensor approach is recent and we can only provide a short time-series based on our preliminary data. The intensity of sampling means, however, that daily, monthly and annual variation is now captured with high levels of precision and for some stations is available in near real time. By combining water sampling with sensor data we also have the ability to cross validate to check the quality of our data and to compare to remote sensing (Fig. 9).

Considering the significant resources required for facilitating sustained and integrated oceanography observations over the long term, additional modeling work has been undertaken to test if the NRS network consists of the minimum number of sites necessary to achieve the appropriate resolution for a national, multi-decadal time series. Modeling of the physical components collected by the NRS array identified that in combination, the nine NRSs effectively monitor the inter-annual variability of the continental shelf circulation in about 80% of the region around Australia [55]. This, along with other studies that have used NRS data in part to investigate oceanographic phenomena, such as the 2011 marine heat wave in Western Australia [39], show at least for some parameters, the NRS exceeds design expectations, monitoring and detecting large scale patterns, events and anomalies rather than just local events. Though, our analysis of phytoplankton and zooplankton indicate that the network has no redundancy for these parameters, with all stations either significantly or highly significantly different from each other for community composition.

Separate to these encouraging results, the stations primary role remains to provide sustained and high quality diverse data sets against which other regional studies can be referenced. Further analysis of observations and modeling studies, however, needs to be undertaken - particularly for chemical and other biological attributes - to confirm the adequacy of the network towards both achieving its stated goals and its ability to answer broader science questions. The NRS network, however, does not exist in isolation and draws real strength from being an integral part of the wider IMOS observing system and being complimentary to other more spatially explicit local studies.

## Supporting Information

S1 File **Instructions for accessing raw data.**
(DOCX)

S2 File **Supporting tables.** Table S1. Summary of data collected by the moored sensors of the National Reference Stations (NRS). Table S2. Summary of data collected by the surface meteorology stations deployed at the Maria Island, Darwin, Yongala and North Stradbroke Island National Reference Stations. Table S3. Summary of data collected by additional acidification and bio-optical sensors of the National Reference Stations. Table S4. Summary of data collected by the biogeochemical sampling program of the National Reference Stations.
(DOCX)
