# Supplementary material for: IMOS National Reference Stations: A Continental-Wide Physical, Chemical and Biological Coastal Observing System
Source: PLoS One. 2014 Dec 17;9(12):e113652. doi: 10.1371/journal.pone.0113652 (PMC4269483; doi:10.1371/journal.pone.0113652)
Supplement: S1 File — Instructions for accessing raw data. (DOCX) [file pone.0113652.s001.docx]

Raw Data Instructions for “**IMOS National Reference Stations: a continental wide physical, chemical and biological coastal observing system”**

Data from the NRS are uploaded to the eMII facility and made available to the public through the IMOS Ocean Portal and the Australian Ocean Data Network (AODN) Portal. To access data first go to: http://imos.aodn.org.au/webportal/. Then select the following links to data for each figure:

- Measured parameter

(Figure 5 a & b) - Physical - Water - Turbidity or Salinity - IMOS - ANMN National Reference Stations - Maria and North Stradbroke Islands - Near real-time water quality

(Fig 6 a) - Biological - Pigments - IMOS - SRS Bio-Optical data base of Australian Waters (SRS-OC-BODBAW) Sub-Facility

(Fig 6b) - Biological - Ocean Biota - Biotic taxonomic identification - IMOS National Reference Station (NRS) - Zooplankton Abundance

(Figure 7 a & b) - Biological - Ocean Biota - Biotic taxonomic identification - IMOS National Reference Station (NRS) - Phytoplankton Abundance and Biovolume

(Figure 7 c & d) - Biological - Ocean Biota - Biotic taxonomic identification - IMOS National Reference Station (NRS) - Zooplankton Abundance

(Figure 8) - Biological - Chlorophyll - IMOS - Australian National Mooring Network (ANMN) Facility - WQM and CTD burst averaged data products

(Figure 9) - Physical - Water - Temperature or Salinity - IMOS - ANMN National Reference Stations - Maria and North Stradbroke Islands - Near real-time water quality

and

- Platform

- ship - self propelled boat - IMOS National Reference Station (NRS) - Salinity, Carbon, Alkalinity, Oxygen and Nutrients (Silicate, Ammonium, Nitrite/Nitrate, Phosphate)

Metadata for used in figure 4 is available at: http://data.aodn.org.au/IMOS/public/ANMN/NRS/NRSYON/BIOGEOCHEM/Field_logsheets/.
