# Supplementary material for: IMOS National Reference Stations: A Continental-Wide Physical, Chemical and Biological Coastal Observing System
Source: PLoS One. 2014 Dec 17;9(12):e113652. doi: 10.1371/journal.pone.0113652 (PMC4269483; doi:10.1371/journal.pone.0113652)
Supplement: S2 File — Supporting tables. Table S1. Summary of data collected by the moored sensors of the National Reference Stations (NRS). Table S2. Summary of data collected by the surface meteorology stations deployed at the Maria Island, Darwin, Yongala and North Stradbroke Island National Reference Stations. Table S3. Summary of data collected by additional acidification and bio-optical sensors of the National Reference Stations. Table S4. Summary of data collected by the biogeochemical sampling program of the National Reference Stations. (DOCX) [file pone.0113652.s002.docx]

### Descriptions of the National Reference Stations (NRS)

#### Long-term NRS

##### Port Hacking

The two water sampling stations at Port Hacking are the longest running coastal oceanography time-series in Australia. Located approximately 3 nm offshore, two further stations were established in 1998 at 25 m and 140 m. A further site at 100 m was chosen for instrumentation in 2008 as part of the expanded NRS system, forming a regional array. The array sits within the productive temperate neritic coastal phytoplankton province and the temperate east marine reserve network. The marine environment off New South Wales is dominated by the presence of the East Australian Current (EAC) and the eddy field it produces. The EAC sporadically stimulates upwelling and associated enhanced productivity resulting in phytoplankton blooms and larvae transport [1]. These warm and cold core eddies, generated by the EAC, periodically encroach on the continental shelf and affect coastal waters. The NRS, along with further regional moorings and water sampling locations established by the New South Wales Integrated Marine Observing System (IMOS) science node, enable the monitoring of climate and biological processes of the EAC separation zone and eddy field.

##### Maria Island

The long-term water sampling station on Tasmania’s east coast, is located approximately 4 nautical miles offshore from Maria Island at 40 m depth and lies within the south-east marine reserve network and the temperate neritic phytoplankton province. The site was instrumented in 2008. The NRS is located in waters that constitute one of the few oceanic connection zones between major ocean basins, with the waters of the Pacific Ocean interchanging with those of the eastern Indian Ocean. This connection is unusual, as the local bathymetry results in most of the exchange occurring across relatively shallow depths [2]. The EAC and an extension of the Leeuwin Current, known as the Zeehan Current, seasonally influence the region with a sharp division between the EAC and Zeehan Current occurring off southeast Tasmania [2]. These two currents are diametrically opposed in their intensity, with the Zeehan Current strongest in winter and the EAC strongest in summer [2]. The complex oceanography of the region results in mixing occurring across multiple currents.

##### Rottnest Island

The long-term water sampling station at Rottnest Island is located approximately 17 nm offshore from Western Australia and 2.5 nm from the island itself, at a depth of 50 m. The station is within an oceanographic and predominately dinoflagellate phytoplankton province typical of the tropical Indian Ocean and provides a reference point for the south-west network of marine reserves. In conjunction with a shelf mooring array located to the north of the station and a mooring array in the nearby Perth Canyon, the station monitors along-shelf and cross-shelf exchanges of water, heat, nutrients, plankton and fish larvae. Circulation in the region is anomalous compared to other currents along eastern ocean margins, which tend to flow towards the equator rather than towards the pole. Flowing strongest during the winter, the Leeuwin Current is forced by a sea-level gradient between the north-west continental shelf and the Southern Ocean [3]. The inter-annual variability of the Leeuwin Current is controlled by the Indonesian Through-flow and is also influenced by El Niño Southern Oscillation (ENSO) signals propagating from the western Pacific Ocean to the north of Western Australia. The prevailing winds and current direction leads to coastal suppression of upwelling along the Western Australian coast throughout most of the year, and consequently sea surface temperatures of up to 4–5° C warmer than upwelling systems at similar latitudes elsewhere on the globe.

#### New/additional NRS

##### Ningaloo

The Ningaloo NRS, which was implemented in 2010, is located 3.5 nm offshore from North West Cape, Western Australia at a depth of 55 m. The station lies within the Ningaloo Marine Park and the greater North West marine reserve network. This site represents an upgrade from a sampling station first implemented by the Australian Institute of Marine Science (AIMS) in 1997 and builds on a contiguous decadal time-series. The NRS is located in a region where the Leeuwin Current becomes well formed and is pushed offshore by southerly winds. These winds generate opposing local coastal currents during the summer, including the Ningaloo Current, which interact with a relatively narrow continental shelf to generate episodic and transient upwelling [4, 5] Populations of large marine fauna, such as the whale shark [6] exploit enhanced productivity associated with these upwelling events. The Ningaloo Current is thought to have profound impacts on the ecology of coral reef communities living among Ningaloo Reef [7] including enhancing local primary productivity and nutrient flow onto the reef [8].

##### Yongala

The Yongala NRS is located approximately 11 nm from shore in the Great Barrier Reef (GBR) lagoon, which lies within the GBR Marine Park and adjacent to the Coral Sea marine reserve network. Situated at a depth of 28 m, the station is well placed to document the competing influences of the south-eastward lagoonal branch of the EAC [9] and the opposing south-easterly trade wind forced coastal current [10] and seasonal intrusions of freshwater plumes which occur in association with monsoonal wet season flooding. Sampling at the station will provide a better understanding of the distinct GBR phytoplankton province, which is dominated by fast growing nanoplankton diatoms. The station complements the larger Queensland IMOS node’s GBR mooring array that delivers data to monitor the major boundary currents along the continental slope and outer shelf-ocean exchanges, a key focus of the node. Given its proximity to the coral reef system of the GBR, and concerns of the effects of ocean acidification on corals, this NRS is equipped with additional instruments to monitor CO_2_ uptake by the ocean and therefore provide data-streams to better understand the effects of a changing pH environment on the GBR.

##### North Stradbroke Island

The North Stradbroke Island NRS is located in 60 m of water, 6.6 nm north east of North Stradbroke Island, within the Moreton Bay Marine Park and adjacent to one of the openings of Moreton Bay. Its location makes it well suited for assessing impacts of the rapidly expanding south-east Queensland population centre on the coastal environment and the temperate east network of marine reserves. The area is also a biogeographic boundary between tropical and subtropical regions for a number of marine species including temperate neritic and GBR lagoon phytoplankton communities. The NRS provides the coastal point on a transect of instrumented monitoring sites that form an array that stretches from within Moreton Bay, across the continental shelf and into the open ocean, facilitated by a collaborative effort between the Australian National Mooring Network (ANMN) and the Australian Bluewater Observing System (ABOS) facilities of IMOS and the Terrestrial Ecosystem Research Network (TERN). The characteristic southward flow of the EAC is clearly observed adjacent to this part of the east Australian coast between 22° and 35°S and it is in this region that the current’s main intensification occurs and the maximum surface currents and volume transport are achieved [11].

##### Kangaroo Island

The most easterly of the three NRS that lie within the south-west marine reserve network, the Kangaroo Island NRS is located 4.8 nm west of Kangaroo Island in South Australia. The station is located in 110 m of water and within the temperate neritic phytoplankton province, and also borders a highly variable transition zone between the embedded tropical flora flowing from the Leeuwin Current and the Subantarctic province. The NRS’s *in situ* sensors are moored just below a steep convergence between the 50 m and 100 m isobath to monitor flows in the region driven by winds, the eastward flowing Leeuwin Current during winter and indirectly, the westward Flinders Current, which in turn drives the Leeuwin Undercurrent. Upwelling occurs during summer in the region, but local winds, ENSO events and possibly canyon-induced upwelling moderate the intensity of this seasonal upwelling [12]. The location of the NRS also allows monitoring of dense water from the adjacent Spencer Gulf which is expelled during winter and the upwelling of deeper waters on the shelf in this region and, hence, the transfer of dissolved inorganic carbon into the shelf region. The NRS compliments a regional mooring array in the area that provides data on the relative importance of local and large-scale forcing on currents, low-frequency flows and thermocline depth in association with climate phenomena and anomalies.

##### Esperance

The Esperance NRS is located 1.5 nm offshore on the southern side of Western Australia at a mid point across the south-west marine reserve network. Located at a depth of 50 m, the station experiences the influence of the Leeuwin Current during the winter and intermittent cold water upwelling from the Southern Ocean in response to surface winds during the summer. This makes Esperance the only NRS within the highly variable oceanic transition province of Australia’s phytoplankton communities. The regions terrestrial and coastal oceanic ecosystems are internationally recognised as contributing to a global hotspot for biodiversity [13, 14]. The southern region of Western Australia is one in which multi-decadal declines in rainfall have been recorded and links between rainfall in the region and both mean sea level pressure and sea-surface temperature are suggestive of coupled air-sea interactions over the southern Indian Ocean which may be relevant at decadal or multi-decadal timescales [15]. Long-term monitoring of the coastal ocean at Esperance therefore provides data vital for understanding the relationship between marine processes, biodiversity and rainfall in this isolated, wet and temperate corner of Australia.

##### Darwin

The Darwin NRS is located in the Beagle Gulf, 4 nm offshore from the Cox Peninsula in the Northern Territory at a depth of 20 m. Its location is characteristic of the shallow coastal seas of Australia’s far north where macro-tides can range up to 8 m and produce strong tidal currents of 2 ms^-1^. Sited within the northern network of marine reserves, the NRS is on the border of the north- west and northern shelf water phytoplankton provinces, which are dominated by tropical diatom flora. The NRS is located in an inverse estuary that imports oceanic water during Austral winter, known in the Australian tropics as the ‘dry season’, resulting in waters that are vertically well mixed. However, during the Austral tropical summer or ‘wet season’, the water column becomes highly stratified across periods from a few days up to a few weeks. As a result of the tidal nature and monsoonal weather patterns, sediment re-suspension dominates the character of the water throughout northern coastal waters and is a key parameter measured by the station.

## References

1. M. Roughan, J.H. Middleton, A comparison of observed upwelling mechanisms off the east coast of Australia, Cont. Shelf Res. 22 (2002) 2551–2572. doi: 10.1016/S0278-4343(02)00101-2.

2. K. R. Ridgway, Seasonal circulation around Tasmania: an interface between eastern and western boundary dynamics, J. Geophys. Res. 112 (2007) C10016. doi:10.1029/2006JC003898.

3. G. R. Cresswell, T. J. Golding, Observations of a south-flowing current in the south eastern Indian Ocean, Deep Sea Res. 27 (1980) 449–66.

4. G. H. Gersbach, C.B. Pattiaratchi, G.N. Ivey, G. R. Cresswell, Upwelling on the south-west coast of Australia – source of the Capes Current?, Cont. Shelf Res. 19 (1999) 363–400. doi:10.1016/S0278-4343(98)00088-0.

5. M. Woo, C. Pattiaratchi, W. Schroeder, Summer surface circulation along the Gascoyne Continental Shelf, Western Australia, Cont. Shelf Res. 26 (2006) 132–152. doi:10.1016/J.CSR.2005.07.007.

6. S.G. Wilson, J.G. Taylor, A.F. Pearce, The seasonal aggregation of whale sharks at Ningaloo Reef, Western Australia: currents, migrations and the El Niño/Southern Oscillation, Enviro. Bio. Fish. 61 (2001) 1–11.

7. J.G. Taylor, A.F. Pearce, Ningaloo Reef currents: implications for coral spawn dispersal, zooplankton and whale shark abundance, J. Roy. Soc. West. Aust. 82 (1999) 57–65.

8. M. Feng, K. Wild-Allen K, The Leeuwin Current, in: K.-K. Liu, L. Atkinson, R. Quiñones, L. Talaue-McManus (Eds.), Carbon and nutrient fluxes in continental margins: a global synthesis, Springer, Berlin, 2010, pp 197–210.

9. R. Brinkman, E. Wolanski, E. Deleersnijder, F. McAllister, W Skirving, Oceanic inflow from the Coral Sea into the Great Barrier Reef, Estuar. Coast. Shelf Sci. 54 (2001) 655–668. doi: 10.1006/ecss.2001.0850.

10. D.M. Burrage, J.A. Church, C.R. Steinberg, Linear systems analysis of momentum on the continental shelf and slope of the central Great Barrier Reef, J. Geophys. Res. 96 (1991) 22169–22190.

11. K.R. Ridgway, J.R. Dunn, Mesoscale structure of the mean East Australian Current system and its relationship with topography, Prog. Oceanog. 56 (2003) 189–222. doi:10.1016/S0079-6611(03)00004-1.

12. J.F. Middleton, J.T. Bye, The physical oceanography of Australia’s southern shelves: a review, Prog. Oceanog. 75 (2007) 1–41. doi:10.1016/j.pocean.2007.07.001.

13. N. Myers, R.A. Mittermeier, C.G. Mittermeier, G.A.B. da Fonseca, J. Ken, Biodiversity hotspots for conservation priorities, Nature 403 (2000) 853–858.

14. J.A. Phillips, Marine macroalgal biodiversity hotspots: why is there high species richness and endemism in southern Australian marine benthic flora?, Biodiversity Conserv. 10 (2001) 1555–1577.

15. I.N. Smith, P. McIntosh, T.J. Ansell, C.J.C. Reason, K. McInnes, Southwest Western Australian winter rainfall and its association with Indian Ocean climate variability, Int. J. Climatol. 20 (2000) 1913–1930. doi: 10.1002/1097-0088(200012)20:15<1913::AID-JOC594>3.0.CO;2-J.

Table S1. Summary of data collected by the moored sensors of the National Reference Stations (NRS)

| Data stream | Format/Units | Depth (m) |
| --- | --- | --- |
| Site | – | ~20, seafloor |
| Time | hh:mm:ss | n/a |
| Date | mm/dd/yy | n/a |
| Conductivity | mmho | ~20, seafloor |
| Temperature | °C | ~20, seafloor |
| Pressure | Dbar | ~20, seafloor |
| Conductivity (salinity) | PSU | ~20, seafloor |
| Dissolved O_2_ | μmol·l^-1^ | ~20, seafloor |
| Oxygen | % | ~20, seafloor |
| Chl a (raw) | Counts | ~20, seafloor |
| Chl *a* | µg l^-1^ | ~20, seafloor |
| Turbidity (raw) | Counts | ~20, seafloor |
| Turbidity | NTU* | ~20, seafloor |
| Velocity | m·s^-1^ | Water column |
| Direction | heading | Water column |
|  |  |  |

*NTU = Nephelometric Turbidity Units

Table S2. Summary of data collected by the surface meteorology stations deployed at the Maria Island, Darwin, Yongala and North Stradbroke Island National Reference Stations

| Data stream | Units | Depth (m) |
| --- | --- | --- |
| Barometric pressure | hPa | -1 |
| Wind speed | m·s^-1^ | -1 |
| Wind direction | 0–360° | -1 |
| Air temperature | °C | -1 |
| Liquid precipitation | mm | -1 |
| Relative Humidity | % | -1 |
| Sea surface temperature | °C | 1 |

Table S3. Summary of data collected by additional acidification and bio-optical sensors of the National Reference Stations

| Data stream | Units | Depth (m) |
| --- | --- | --- |
| pCO_2_ | ppm | Surface |
| DO | umol·kg^-1^ | Surface |
| Temperature | °C | Surface |
| Conductivity (salinity) | (PSU) | Surface |
| CDOM | mg·m^-3^ | 20 |
| Blue wavelength | mg·m^-3^ | 20 |
| Green wavelength | mg·m^-3^ | 20 |
|  |  |  |

Table S4. Summary of data collected by the biogeochemical sampling program of the National Reference Stations

| Data stream type | Format/Units | Depth (m) |
| --- | --- | --- |
| Site | – | n/a |
| Time | hh:mm:ss | n/a |
| Date | dd/mm/yyyy | n/a |
| Secchi disk depth | m | n/a |
| Conductivity | mmho | 0-2.5m (profile) |
| Temperature | °C | 0-2.5m (profile) |
| Depth | M | 0-2.5m (profile) |
| Chl *a* | µg l^-1^ | 0-2.5m (profile) |
| Turbidity | NTU | 0-2.5m (profile) |
| Dissolved oxygen | μmol·l^-1^ | 0-2.5m (profile) |
| Total CO_2_ | μmol·kg^-1^ | 4–7 depths at each site |
| Total alkalinity | μmol·kg^-1^ | 4–7 depths at each site |
| pH ^a^ | – | 0, 10, 20, 30, 40, 50 |
| Nitrites/Nitrate ^a^ | μmol·l^-1^ | 0, 10, 20, 30, 40, 50 |
| Nitrite ^a^ | μmol·l^-1^ | 0, 10, 20, 30, 40, 50 |
| Silicates ^a^ | μmol·l^-1^ | 0, 10, 20, 30, 40, 50 |
| Orthophosphate ^a^ | μmol·l^-1^ | 0, 10, 20, 30, 40, 50 |
| Ammonia ^a^ | μmol·l^-1^ | 0, 10, 20, 30, 40, 50 |
| Salinity ^a^ | PSU | 0, 10, 20, 30, 40, 50 |
| Dissolved oxygen ^a b^ | μmol·l^-1^ | 0, 10, 20, 30, 40, 50 |
| Phytoplankton taxon abundance | Species(Cells) L^-1^ | Water column |
| Phytoplankton taxon group ^c^ | 1,2,3,4 | Water column |
| Phytoplankton biomass | ml·l^-1^ | Water column |
| HPLC pigments | µg·l^-1^ (or mg·m^-3^) | Water column |
| Flow cytometry | Cell·l^-1^ | Water column |
| Total suspended matter | mg·l^-1^ | Water column |
| Organic | mg·l^-1^ | Water column |
| Inorganic | mg·l^-1^ | Water column |
| Zooplankton dry weights | mg·m^-3^ | Water column |
| Zooplankton taxon group | Copepod/other | Water column |
| Zooplankton phylogeny | Phylum/Order/Class | Water column |
| Zooplankton species | Genera/Species | Water column |
| Zooplankton taxon abundance | Species number/m^-3^ | Water column |
| Zooplankton growth stage | Adult/Juvenile/Naupilus | Water column |
| Zooplankton sex | Male/female | Water column |

^a^Except Darwin and Yongala, where depth <50m.

^b^Laboratory analysis presented is for Maria Island.

^c^1: diatoms; 2: dinoflagulates; 3:small flagellates; 4: cyanobactreria.
